# Agreement between parental reports and accelerometer measures of sleep duration in primary school children

**DOI:** 10.1038/s41598-025-07786-w

**Published:** 2025-07-15

**Authors:** Ana Duarte, Juliana Martins, Rafaela Rosário

**Affiliations:** 1 Health Sciences Research Unit: Nursing (UICISA: E), Nursing School of Coimbra, Avenida Bissaya Barreto, Polo C, Coimbra, 3046-851 Portugal; 2 School of Nursing, University of Minho, Edifício 4, Campus de Gualtar, Braga, 4710-057 Portugal; 3 Research Centre on Nursing (CiEnf), School of Nursing, University of Minho, Edifício 4, Campus de Gualtar, Braga, 4710-057 Portugal

**Keywords:** Sleep, Sleep duration, Children, Agreement analysis, Accelerometry, Self-report, Public health, Epidemiology

## Abstract

Sleep plays a critical role in children’s health and development, with sleep duration being one of the most commonly analyzed measures by researchers. Accurate assessment of sleep duration in school-aged children is crucial for understanding its impact on cognitive development, behavior, and overall health. However, discrepancies between parent-reported sleep duration and objective measurements, such as accelerometry, pose challenges in assessing sleep patterns in children. This study examines the agreement between accelerometer-measured and parent-reported sleep duration in school-aged children from socially vulnerable contexts. A total of 735 children from primary schools wore accelerometers for seven consecutive days, while parents reported their children’s sleep duration. The results revealed significant discrepancies, with parents generally overestimating sleep duration and demonstrating poor predictive accuracy. These findings underscore the need for more reliable methods in assessing sleep duration in children from socially vulnerable backgrounds. By highlighting the discrepancy between accelerometer data and parent reports, the study emphasizes the importance of objective measurements in sleep research. The study’s implications are particularly relevant for public health initiatives aimed at promoting healthy sleep habits among children in socioeconomically disadvantaged communities.

## Introduction

Sleep is a fundamental component of health and well-being, influencing cognitive function, physiological processes, emotional regulation, physical growth, and overall quality of life^1^. Adequate sleep duration varies throughout life. For school-aged children, the recommendation is 9 to 11 h of uninterrupted sleep per night, with consistent bedtimes and wake-up times^1,2^. Numerous studies have shown that chronic insufficient sleep, poor sleep quality, and irregular sleep routines in primary school-aged children are risk factors for various health issues, including impaired cognition, difficulties in emotional regulation, other psychosocial challenges, overweight/obesity, and health conditions such as myopia^3–8^. Specifically, when examining socio-vulnerable contexts, we observe a negative association between socioeconomic status and sleep duration^9,10^.

In line with these, an accurate measurement of sleep is crucial for understanding sleep patterns. In school-aged children, the most commonly used methods to assess sleep duration are parent-reported questionnaires and accelerometer-based evaluations^3,9,11,12^. Both measurement methods are valid and have their advantages and disadvantages. For instance, self-reported or parent-reported questionnaires remain the most practical and cost-effective option for epidemiological studies aiming to gather data from large population-based samples^3,13,14^. However, the presence of both random error and systematic bias in self-reported sleep duration has been noted^3,15^.

Conversely, objective methods like accelerometry have increasingly been utilized to assess 24-hour movement, including physical activity, sedentary behavior, and sleep^16^. These devices are recommended by organizations such as the American Academy of Sleep Medicine as an accurate method for estimating sleep parameters^13,17^. However, collecting device-based measures in large-scale surveillance or time-sensitive examinations is often not feasible, particularly in low- to middle-income countries, due to significant costs and the logistical challenges associated with device use^14^.

Due to the characteristics of these measurement methods and considering that sleep has been extensively studied in numerous research projects and will continue to be in many others, we prioritize understanding the best practices for assessing sleep duration in a specific group characterized by social vulnerability and poverty, particularly whether one method is preferable or if both methods are effective.

Therefore, the aim of this study was to analyze the agreement between parent-reported and objectively measured sleep duration in school-aged children from socially vulnerable contexts.

## Methods

### Study design and ethics

This is a cross-sectional study, which is part of a broader research, BeE-school Project, conducted in ten primary schools from the north of Portugal. Informed consent was obtained from participants and their legal guardians. A total of 735 school-aged children participated and were enrolled in the study.

Inclusion criteria included children from ten primary schools situated in economically and socially disadvantaged areas characterized by poverty and social exclusion. The study excluded children with cognitive or physical impairments that might interfere with data collection.

Due to the social context of the present study, parents’ education levels were collected via a questionnaire and categorized as less than secondary education, secondary education, or higher education (university level). In the descriptive data, we chose to include the highest education level attained by either parent.

The BeE-school Project obtained ethical approval from the Ethics Committee for Life and Health Sciences at the University of Minho (CEICVS 009/2022).

The research adhered to the Code of Ethics of the World Medical Association (Declaration of Helsinki).

### Outcome assessments

In this study, the sleep period refers to the opportunity to sleep. It includes the total time between going to bed and waking up, as well as the wake time immediately before and after falling asleep. This definition has been used in other works, such as Nauha and colleagues^18^ who considered wakefulness occurring before and after a major sleep episode as part of the sleep period^18^.

Data collection occurred in autumn/winter between months of October and December 2022 and included parent-reported sleep and objectively measured sleep using accelerometers.

#### Parent-reported sleep duration

Parents reported children’s usual bedtime and wake-up time on weekdays and weekends through four additional questions included in the Children’s Sleep Habits Questionnaire, a retrospective questionnaire answered by parents that assesses sleep behavior in school-aged children^19,20^. The total sleep duration was calculated by subtracting the bedtime from the wake-up time (i.e., wake-up time − bedtime).

Sleep duration was categorized according to Hirshkowitz and colleagues’^1^ recommendations into agree/not agree with the recommendations (agree if sleep duration is between 9 and 11 h per day for school-aged children).

#### Objective assessment of sleep duration

Children were instructed to wear the triaxial Actigraph wGT3X-BT accelerometer (Actigraph LLC, Pensacola, FL, USA) in the dominant wrist continuously for 24 h a day over a span of 7 days, except during water-related activities such as bathing or swimming, or during organized sports where accelerometer use is not allowed. Accelerometer device was placed on the dominant wrist. Dominant wrist was chosen instead non-dominant in order to enhance adherence, comfort, and compliance. Also, this positioning allows to facilitate the verification process for professors regarding the use of the devices, as it makes them more visible if they are writing with this hand. Furthermore, placing the accelerometer on the dominant wrist is generally more comfortable for participants, enhancing compliance and ensuring more reliable data collection, as participants are less likely to remove or adjust the device. Additionally, some participants habitually wear watches on their non-dominant wrist and preferred to maintain this routine. According to Dieu and colleagues^21^there is no notable variances in physical activity outcomes between accelerometers worn on the dominant and non-dominant wrists, regardless of the axis. Driller and colleagues^22^ also found no significant differences between actigraphy on the non-dominant and dominant wrists for monitoring sleep.

After retrieval, the accelerometers were subjected to data downloading, followed by data preparation for subsequent analysis. Data were excluded if accelerometer wear time fell below 16 h per day across a minimum of three weekdays and two weekend day. Non-wear periods were estimated based on the standard deviation and value range of each accelerometer axis, using a 60-minute window with 15-minute increments^23^.The Actigraph wGT3X-BT accelerometers were initialized and managed using Actilife software (version 6.13.5, Actigraph LLC). They were set to capture data at a frequency of 60 Hz, commencing data collection at 10am on the day the device was applied. The stop time was scheduled for 10am on the retrieval day. Data were retrieved and stored in raw.gt3x format for further processing. The raw accelerometer data were reviewed to identify potential outliers, particularly concerning sleep timing and consistency.

The research team conducted on-site visits to schools in order to install the accelerometer devices on the participants. In addition, they supplied detailed instructions and documentation sheets to record pertinent information regarding the children’s daily activities and sleep patterns from the preceding night, including instances of non-wear and specific bedtime and wake-up times. Subsequently, after a span of one week, the research team returned to the schools to retrieve the accelerometers.

Sleep duration was estimated using van Hees et al.’s method integrated into GGIR processing, employing the HDCZA algorithm to identify sustained wrist inactivity characterized by a lack of change in the wrist z-angle by more than five degrees for at least five minutes. This algorithm utilizes a guiding window derived from a sleep log or algorithmic estimate to determine sleep periods. It first identifies prolonged periods of arm angle stability and selects the longest merged period as the guiding window^24^.

Data processing was done in R (Version 4.3.2) using the R-package GGIR (version 3.0.1)^25^. This software autonomously adjusts the raw triaxial accelerometer signals and transforms them into vector magnitude units, correcting for gravity. This transformation is referred to as the Euclidean Norm Minus One (ENMO), calculated as the square root of the sum of the squares of the three axis minus1g(ENMO = x2 + y2 + z2 − 1 g).

As in parent-reported data, sleep duration was categorized according to Hirshkowitz and colleagues’^1^ recommendations into agree/not agree with the recommendations (agree if sleep duration is between 9 and 11 h per day for school-aged children).

### Statistical analysis

The descriptive variables were calculated for all participants and separately for boys and girls. Descriptive analysis included central tendency measures and dispersion according to the type of variable. For a descriptive analysis of the quantitative variables, the mean and standard deviation (mean ± SD) were calculated; for categorical variables, count and percentages (n (%)) were used. We also compared the proportions of categorical variables using chi-squared tests and Student’s *t*-test.

To analyze the agreement between objectively measured sleep duration through accelerometers and parent-reported information, we conducted four distinct analyses: calculation of the intraclass correlation coefficient (ICC), Bland-Altman plots, Cohen’s kappa, and receiver operating characteristic (ROC) curves, along with additional calculations of positive predictive value (PPV) and negative predictive value (NPV). We selected these four statistical analyses as they complement each other, providing a comprehensive assessment of agreement. The Bland-Altman method determines the limits of agreement (LoA) but does not establish a predefined threshold for an acceptable degree of agreement^26^. The Intraclass Correlation Coefficient (ICC) serves as a reliability index, reflecting both correlation and agreement between measures^27^. Cohen’s kappa evaluates inter-rater agreement, offering insight into categorical data consistency^28,29^. Lastly, Receiver Operating Characteristic (ROC) curves, through the Area Under the Curve (AUC), allow for the comparison of overall accuracy between different tests assessing the same condition^30^.

^262728,2930^A level of significance of 0.05 was considered for all analyses. The software IBM SPSS^®^ (version 29.0.1.1) was used for all statistical computations.

#### Intraclass correlation coefficient

Intraclass correlation coefficient (ICC) between reported and objectively sleep duration measures was calculated. The ICC is commonly used to quantify the reliability or agreement between two or more measurements, particularly when assessing continuous data. In this study, we were interested in evaluating the degree of absolute agreement between two specific measurement methods — subjective reports from parents and objective recordings from accelerometers.

To determine the appropriate form of ICC, we followed the framework outlined by Koo and Li^27^which suggests selecting the ICC form based on three key factors: the model (one-way vs. two-way), the type of measurement (single vs. average), the definition (consistency vs. absolute agreement). Given that both parent-reported and accelerometer-measured sleep durations were available for each child, we employed a two-way mixed effects model. This model was chosen because the two sources of measurement (parents and accelerometers) were considered fixed, as they represented specific methods rather than a random sample of raters from a larger population.

To examine the agreement between the two methods of measurement on an individual basis (rather than the average of multiple measurements), we calculated the ICC for single measures. This approach is appropriate when the goal is to assess the reliability of each individual measurement method rather than the mean of multiple raters or instruments. We applied the ICC using the absolute agreement definition, as the focus was on evaluating how closely the two measurement methods matched in terms of absolute values. Specifically, we aimed to determine whether parent-reported sleep duration and accelerometer-measured sleep duration agreed on a one-to-one basis, rather than merely assessing the consistency in ranking or relative differences between the two methods.

The results were interpreted based on the following scale: ICC values less than 0.50 indicate poor agreement, values between 0.50 and 0.75 indicate moderate agreement, and values greater than 0.75 indicate good to excellent agreement^27^.

#### Bland and Altman analysis

In addition to the ICC, we conducted a Bland-Altman analysis to further assess the agreement between parent-reported and accelerometer-measured sleep durations. The Bland-Altman method is a well-established statistical approach for evaluating the agreement between two quantitative measurement methods by analyzing the differences between paired measurements^30,31^. It offers a detailed exploration of the data by allowing for the visualization of the magnitude of differences between the two methods across the range of values^26^.

The Bland-Altman method consists of plotting the mean of the two measurements against their difference. The mean difference, standard deviation of the differences, and 95% LoA were calculated and represented in Bland-Altman plots^31^. Specifically, we calculated the average of the differences between the two methods across all subjects, representing the systematic bias between parent-reported and accelerometer-measured sleep duration. A positive mean difference indicates that, on average, parents overestimate sleep duration, whereas a negative mean difference suggests underestimation.

To assess the variability in the differences, we calculated the LoA, defined as the mean difference ± 1.96 times the standard deviation of the differences^31^. These LoA help determine whether the agreement between the two methods is acceptable within the context of the study^30^. The mean difference and the 95% LoA allowed us to evaluate whether parent-reported sleep duration could be considered a valid proxy for the objectively measured duration by accelerometers.

#### Cohen’s kappa

Cohen’s Kappa coefficient was employed to assess inter-rater reliability and quantify the agreement between objective (accelerometer-based) and parent-reported sleep duration, while accounting for agreement due to chance. Kappa values were interpreted using the following scale: 0.01–0.20 indicating slight agreement, 0.21–0.40 as fair agreement, 0.41–0.60 as moderate, 0.61–0.80 as substantial, and 0.81–1.00 as almost perfect agreement. For all Kappa values, 95% confidence intervals were calculated^32^.

#### Receiver operating characteristic (ROC) curve

To assess the predictive ability of parent-reported sleep duration compared to objective accelerometer-based measurements, ROC curves were utilized with accelerometer data serving as the gold standard. Sensitivity and specificity were calculated to evaluate the accuracy of parent reports against accelerometer readings. Sensitivity refers to the proportion of subjects with the condition who are correctly identified by the test, while specificity is the proportion of subjects without the condition who are correctly identified by the test^30^. The AUC was calculated to assess overall predictive performance, with an AUC of 1.0 indicating perfect prediction and 0.5 suggesting no predictive value^30^.

Additionally, to evaluate the validity of parent-reported sleep duration in comparison with accelerometer recordings, we calculated positive predictive value (PPV) and negative predictive value (NPV). PPV represents the proportion of subjects with a positive test result who actually have the condition, whereas NPV represents the proportion of subjects with a negative test result who do not have the condition^30^.

## Results

Table 1 presents descriptive data. The study included 735 children (380 boys and 355 girls), with a mean ± SD age of 7.7 ± 1.2 years.

According to parent-reported information, children sleep had a mean ± SD of 9:50 ± 0:47 h on weekdays, with 90.9% of children following the recommendations for sleep duration. On weekend days, children sleep had a mean ± SD of 10:25 ± 0:53 h, with 81.2% following the recommendations.

According to objectively measured sleep duration through accelerometers, children sleep had a mean ± SD of 8:57 ± 0:43 h on weekdays, with just approximately a half (49.2%) of them following the recommendations for sleep duration. On weekend days, children sleep had a mean ± SD of 9:15 ± 1:03 h, with 61.7% following the recommendations.

**Table 1:** Descriptive data.

	Total (735)	Boys (380)	Girls (355)	*p*-value
Age (years)	7.7 ± 1.2	7.7 ± 1.2	7.7 ± 1.2	0.665
Parent’s highest education level				0.220
Less than secondary	82 (13.4)	35 (11.4)	47 (15.6)	
Secondary	224 (36.7)	111 (36.0)	113 (37.4)	
University studies	304 (49.8)	162 (52.6)	142 (47.0)	
Sleep duration (parent-reported) - hours				
Weekdays	9:50 ± 0:47	9:51 ± 0:46	9:50 ± 0:47	0.947
Weekends	10:25 ± 0:53	10:18 ± 0:52	10:33 ± 0:54	0.002
Sleep recommendations (parent-reported) – weekdays				0.247
Follow the recommendations	491 (90.9)	253 (92.3)	238 (89.5)	
Don’t follow the recommendations	49 (9.1)	21 (7.7)	28 (10.5)	
Sleep recommendations (parent-reported) – weekends				0.297
Follow the recommendations	419 (81.2)	219 (83.0)	200 (79.4)	
Don’t follow the recommendations	97 (18.8)	45 (17.0)	52 (20.6)	
Sleep duration (actigraphy) - hours				
Weekdays	8:57 ± 0:43	8:56 ± 0:43	8:57 ± 0:43	0.818
Weekends	9:15 ± 1:03	9:11 ± 1:00	9:26 ± 0:56	0.003
Sleep recommendations (actigraphy) – weekdays				0.595
Follow the recommendations	286 (49.2)	143 (48.1)	143 (50.4)	
Don’t follow the recommendations	295 (50.8)	154 (51.9)	141 (49.6)	
Sleep recommendations (actigraphy) – weekends				0.008
Follow the recommendations	303 (61.7)	132 (55.7)	171 (67.3)	
Don’t follow the recommendations	188 (38.3)	105 (44.3)	83 (32.7)	

Note: values are expressed as mean ± SD for continuous variables and n (%) for categorical ones.

The agreement analysis using ICC revealed low values (0.189 for weekdays and 0.139 for weekends), indicating that the two measurements (parent-reported and accelerometer-measured sleep duration) are not in agreement, as shown in Table 2. This discrepancy may reflect that parents tend to either underestimate or overestimate their children’s sleep duration compared to the more objective measurement. Despite the significant p-value, the low agreement between the methods suggests that the parents’ reports are not a reliable substitute for accelerometers in terms of sleep duration accuracy.

**Table 2:** Analysis of agreement between parent-reported and actigraphy-assessed sleep duration in children, using the intraclass correlation coefficient (ICC).

	ICC	95% Confidence interval
Parent-reported × actigraphy-assessed on weekdays	0.189	(−0.043, 0.387)
Parent-reported × actigraphy-assessed on weekend days	0.139	(−0.045, 0.309)

All ICC *p*-values < 0.001.

**Fig. 1:**
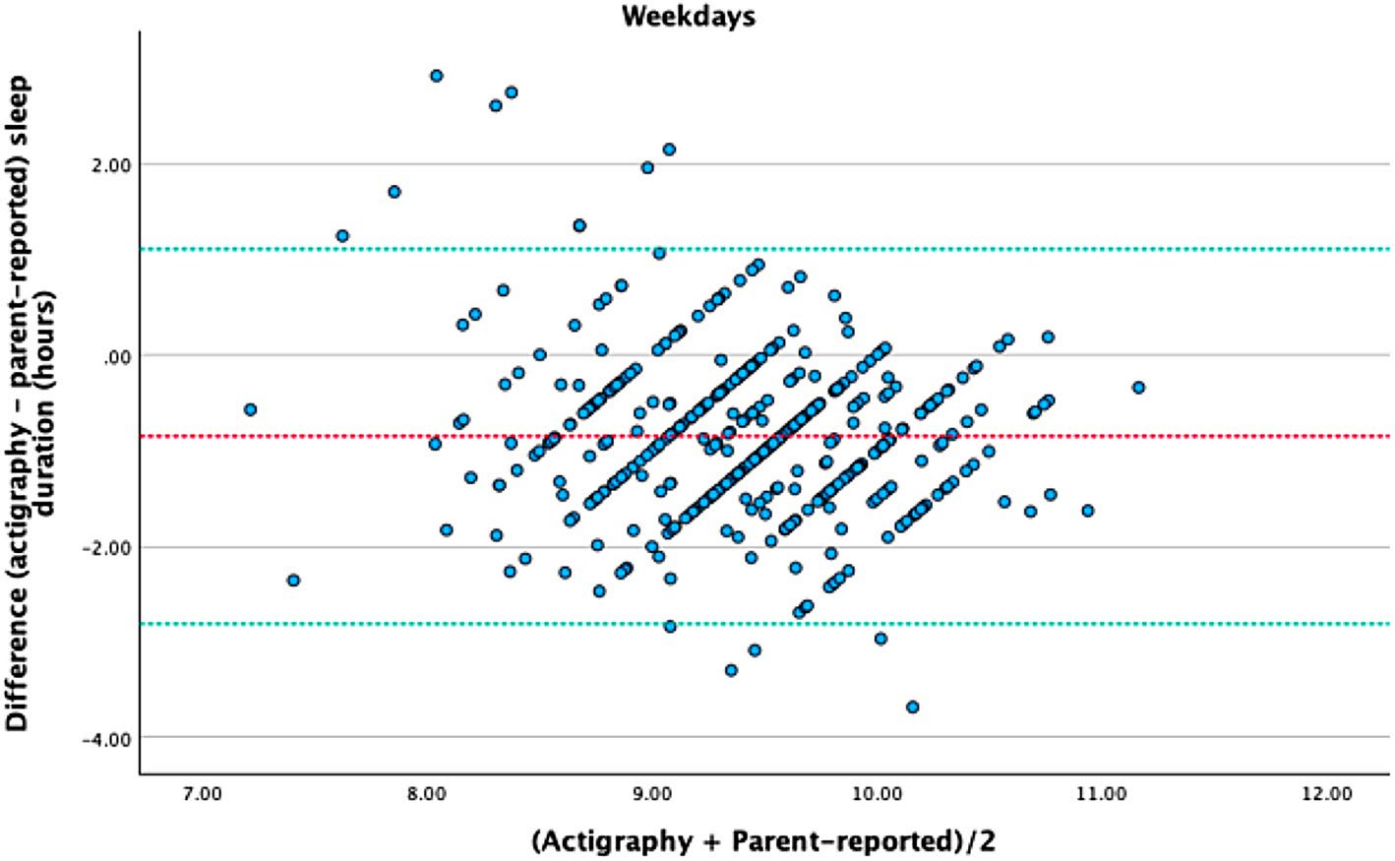
Bland-Altman plots (mean difference and 95% LoA (lower and upper) for objectively measured and parent-reported children’s sleep duration) on weekdays.

**Fig. 2:**
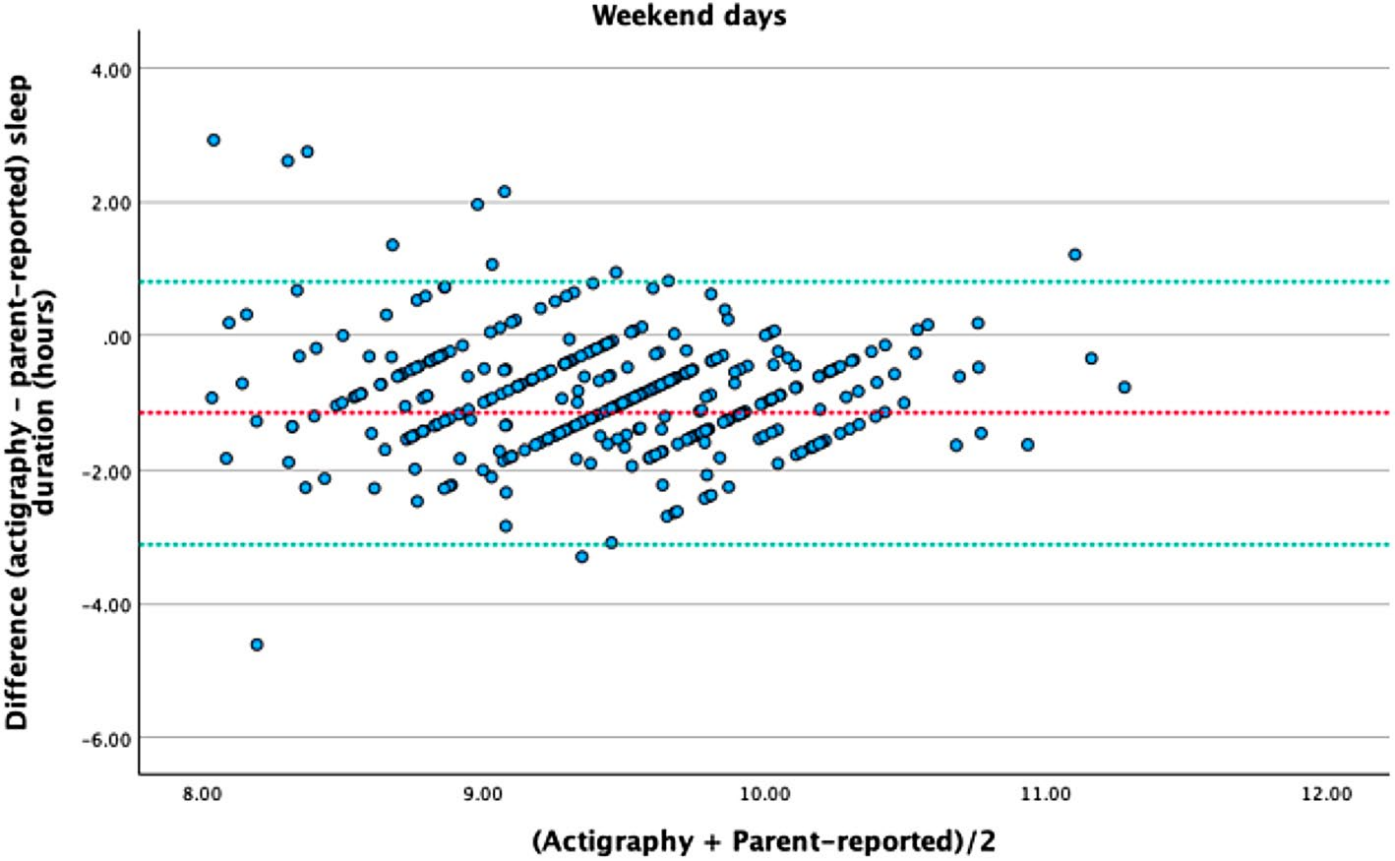
Bland-Altman plots (mean difference and 95% LoA (lower and upper) for objectively measured and parent-reported children’s sleep duration) on weekend days.

To better understand the results, we performed a Bland-Altman analysis to assess the agreement between accelerometer-measured and parent-reported children’s sleep duration on weekdays and weekends (see Figs. 1 and 2). The mean ± SD difference between accelerometer-measured and parent-reported sleep durations was − 0.85 ± 0.83 h on weekdays, and − 1.15 ± 1.09 h on weekends. This suggests that parents tend to overestimate their child’s sleep duration compared to the objective measurements.

The 95% LoA were calculated as −2.81 to 1.11 h on weekdays. This range suggests that, for most children, the difference between accelerometer-measured and parent-reported sleep durations on weekdays will fall within this interval. In practical terms, this means that parents may overestimate sleep duration by as much as 2.81 h or underestimate it by up to 1.11 h compared to accelerometer measurements. The relatively wide range of these limits indicates considerable variability in the degree of parental estimation during the week.

For the weekend, the 95% LoA were slightly wider, ranging from − 3.11 to 0.81 h. This indicates greater variability in the differences between the two methods during weekends, with parents potentially overestimating sleep duration by up to 3.11 h or underestimating it by as much as 0.81 h compared to accelerometer measurements.

After dichotomizing sleep duration into meeting or not meeting the recommendations for school-aged children, a contingency table was created to assess the distribution of participants who met or did not meet the recommended sleep guidelines according to both accelerometer measurements and parent reports (Tables 3 and 4). This table provides an overview of the agreement and disagreement between the two methods, offering additional insight into the classification accuracy of parent-reported sleep duration compared to objective measurements.

**Table 3:** Contingency table comparing the classification of children as meeting or not meeting the recommended sleep duration according to parent-reported and accelerometer-measured sleep on weekdays.

Weekdays				
		Parent-reported		Total
		Meeting	Not meeting	
Accelerometer (Gold standard)	Meeting	**229**	**20**	**249**
	Not meeting	**205**	**23**	**228**
Total		**434**	**43**	**477**

**Table 4:** Contingency table comparing the classification of children as meeting or not meeting the recommended sleep duration according to parent-reported and accelerometer-measured sleep on weekend days.

Weekend days				
		Parent-reported		Total
		Meeting	Not meeting	
Accelerometer (Gold standard)	Meeting	**187**	**55**	**242**
	Not meeting	**140**	**25**	**165**
Total		**327**	**80**	**407**

We conducted a Cohen’s Kappa analysis to assess the agreement between objectively measured and parent-reported sleep duration for weekdays and weekend days. The Kappa statistic for the agreement between accelerometer-measured and parent-reported sleep duration on weekdays was 0.021 (*p* = 0.434), indicating a negligible level of agreement. This suggests little to no concordance between the two methods in assessing children’s sleep duration during the week. The Kappa statistic for the agreement between accelerometer-measured and parent-reported sleep duration on weekends was − 0.083 (*p* = 0.059), indicating a level of agreement worse than chance. This suggests substantial discrepancies between the two methods in assessing children’s sleep duration during the weekend.

According to the data presented in the contingency tables, we calculated the sensitivity, specificity, positive predictive value (PPV), and negative predictive value (NPV) for both weekdays and weekend days as follows:

### Weekdays

Sensitivity: 229/249 ≈ 0.920 (92.0%).

Specificity: 23/228 ≈ 0.101 (10.1%).

PPV: 229/434 ≈ 0.528 (52.8%).

NPV: 23/43 ≈ 0.535 (53.5%).

### Weekend days

Sensitivity: 187/242 ≈ 0.773 (77.3%).

Specificity: 25/165 ≈ 0.152 (15.2%).

PPV: 187/327 ≈ 0.572 (57.2%).

NPV: 25/80 ≈ 0.313 (31.3%)

According to these values, we observe that the sensitivity of parent-reported sleep duration is relatively high on weekdays (92.0%), indicating that parents are generally effective at identifying children who meet the recommended sleep guidelines. However, the specificity is quite low (10.1%), suggesting that a high number of children who do not meet the guidelines are incorrectly classified as meeting them. Conversely, on the weekend days, the sensitivity decreases to 77.3%, while the specificity remains low at 15.2%.

The PPV values suggest that when parents report that their child meets the sleep recommendations, there is only a moderate chance (52.8% on weekdays and 57.2% on weekends) that this classification is accurate. Conversely, the NPV values indicate a higher degree of reliability when parents report that their child does not meet the recommendations (53.5% on weekdays and 31.3% on weekends).

To further enhance our agreement analysis, we proceeded to evaluate the ROC curves. This analysis provides additional insights into the diagnostic performance of parent-reported sleep duration in identifying children who meet the recommended sleep guidelines compared to the accelerometer measurements.

The ROC curve analysis yielded an AUC of 0.56 (95% CI: 0.44–0.67), indicating a limited ability of parent-reported sleep duration to predict whether children meet the recommended sleep guidelines compared to accelerometer-measured sleep on weekdays (see Fig. 2). However, this result was not statistically significant (*p* = 0.328). This finding suggests that parent reports demonstrate very weak agreement with objective sleep measurements in classifying children as meeting or not meeting the sleep recommendations on weekdays.

**Fig. 3:**
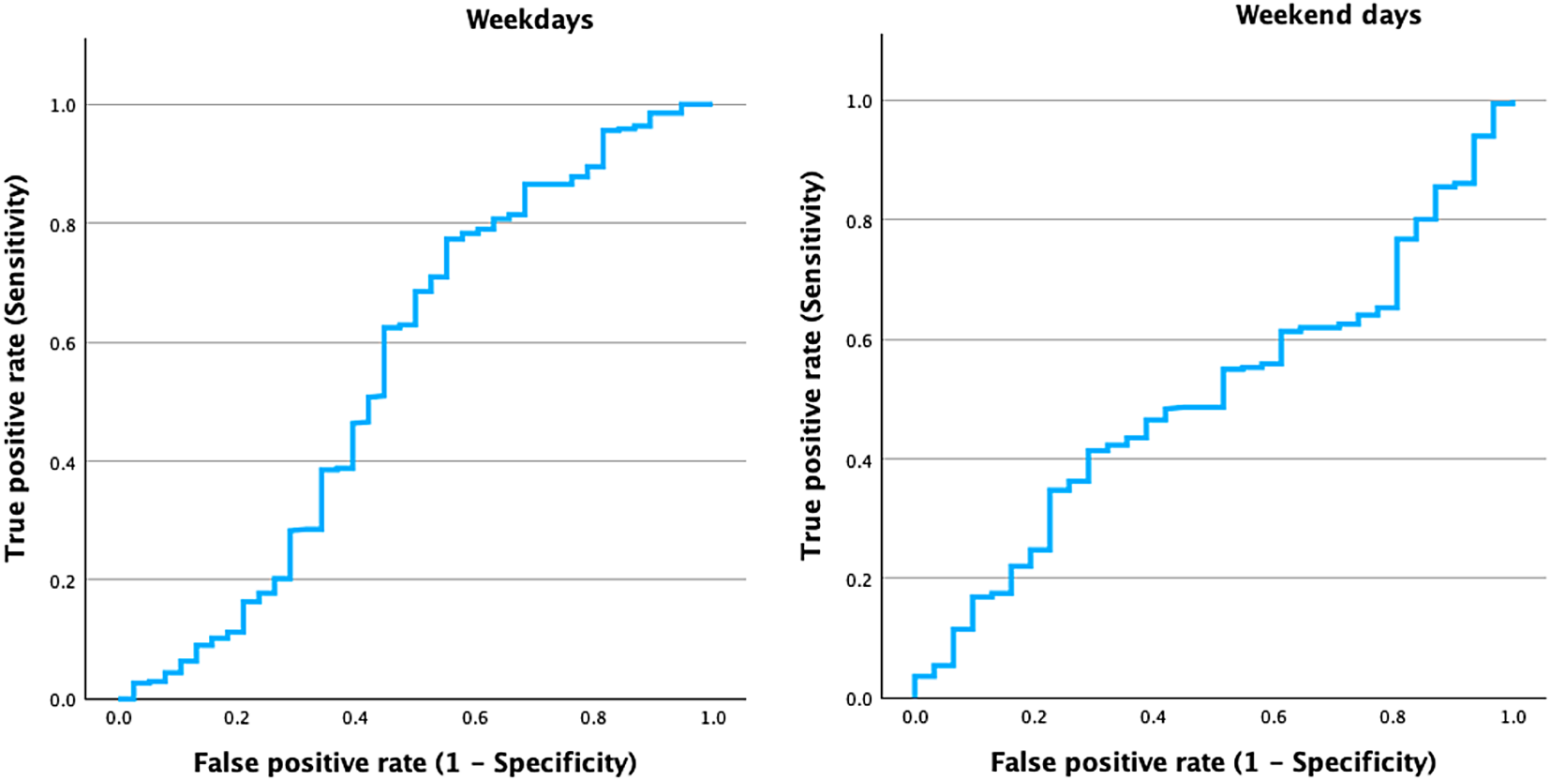
ROC curves evaluating the agreement between accelerometer-measured and parent-reported sleep duration on weekdays and weekend days.

The ROC curve analysis for the weekend yielded an AUC of 0.50 (95% CI: 0.41–0.60), indicating that parent-reported sleep duration has no ability to predict whether children meet the recommended sleep guidelines when compared to accelerometer-measured sleep (see Fig. 3). However, this result did not remain significant (*p* = 0.962). This result suggests that there is virtually no agreement between parent reports and objective measurements in classifying children as meeting or not meeting the sleep recommendations on weekends.

Our findings consistently indicate a lack of agreement between parent-reported and accelerometer-measured sleep duration. This discrepancy underscores the limitations of subjective reports in accurately estimating children’s sleep duration, particularly in socially vulnerable contexts. These results highlight the importance of incorporating objective sleep assessment methods in research and public health initiatives to obtain a more precise understanding of children’s sleep patterns and their implications for health.

## Discussion

This study reveals several key findings in the analyses of the agreement between two methods of measuring sleep duration in school-aged children: parent-reported assessments and actigraphy. There is a significant lack of agreement between accelerometer measurements and parent reports of sleep duration in children.

The ICC values indicate a low level of agreement between the two measurement methods, suggesting considerable variability in the accuracy of parent-reported sleep duration compared to objective measurements. The Bland-Altman plots show that, on average, parents tend to overestimate their children’s sleep duration relative to accelerometer data. The kappa values further highlight the lack of agreement, indicating negligible to poor concordance, which suggests that parent reports do not consistently align with objective measurements. Despite this, the metrics reveal that parents are generally effective at identifying children who meet the recommended sleep guidelines. However, the specificity is very low, indicating a high rate of false positives. The PPV is moderate, while the NPV is higher on weekdays than on weekends. This indicates that although parents are somewhat successful in recognizing which children meet sleep recommendations, the likelihood of misclassification remains a concern, particularly during the weekend. The ROC curve analysis suggests no better than chance performance in predicting whether children meet the recommended sleep guidelines on weekends, while showing a poor ability of parent-reported sleep duration to accurately distinguish between those who do and do not meet the guidelines during weekdays.

Some studies have compared actigraphy with self-reported sleep duration in adults^15,17,33^. For instance, Al Lawati and collaborators^17^ aimed to investigate the agreement between self-reported sleep questionnaires and actigraphy in adults. They concluded that there was a moderate level of agreement between the two methods and emphasized the importance of using objectively measured sleep duration for more accurate results^17^.

Zhenya and Ling^16^ conducted a similar study with preschool children, involving a total of 54 participants with a mean age of 4.6 years. They concluded that, although questionnaires are a useful tool for assessing sleep duration, they may not align as closely with objective measures as other methods do^16^.

Our sample consists of children from socioeconomically vulnerable contexts, which are known to have specific sleep patterns. Studies indicate that sleep problems are more prevalent among individuals from low socioeconomic backgrounds, while children from families with greater parental resources tend to have better sleep quality^10,34–36^. Afonso et al.^9^ in a study conducted in a Portuguese municipality, found that socioeconomic determinants have a significant influence on sleep duration. Factors such as distance from school, daytime and evening activities, schedules, and sleep disorders are closely linked to socioeconomic conditions and have a substantial impact on children’s health^9,37^. Additionally, Rodrigues et al.^37^ highlighted that bedroom use of media devices is more common among children from low-income families, further disrupting nighttime sleep.

In most existing studies to date, involving both adults and children, there is a tendency for self-reports or parent overestimate sleep duration compared to objectively measured data^16,33,38^. This discrepancy may be attributed to social desirability bias, where individuals tend to provide answers they believe are more socially acceptable. Furthermore, socioeconomic status, particularly education level, also contributes to the tendency to provide socially desirable responses^39^.

This study presents several strengths that should be highlighted. First, it examines the agreement between methods in a specific age group that has been relatively understudied. Additionally, it incorporates diverse analytical approaches, providing a comprehensive understanding of the association between the two methods used to measure sleep duration. The findings offer valuable insights into which method may be preferred for assessing children’s sleep duration, whenever feasible.

This study also has some limitations, which are outlined below: this study is its cross-sectional design, which restricts our ability to determine the directionality or causality of the observed relationships. Additionally, the specific context of social vulnerability underscores the need for caution when generalizing our findings to broader populations. This may influence both sleep patterns and the accuracy of parental reporting. To account for this, we used the highest parental education level as a proxy for social context^40,41^. Notably, our findings reveal that nearly half of the parents in the study had a higher education level, which may help mitigate potential biases associated with the social vulnerability context. Another limitation that could be mentioned is the fact that its exclusive focus on sleep duration, without considering other important sleep parameters such as bedtime and wake-up time. While sleep duration is a crucial determinant of child health^42^ other aspects of sleep patterns also play a significant role. For instance, children with Attention-Deficit/Hyperactivity Disorder (ADHD) tend to have later bedtimes and shorter sleep duration compared to their peers^43,44^. A more comprehensive assessment of sleep variables could provide deeper insights into the relationship between socioeconomic factors and sleep health in socially vulnerable populations. Additionally, while accelerometry provides objective data on sleep duration, it has limitations in capturing sleep quality and other nuanced sleep parameters^45^. Furthermore, inaccuracies may arise if children improperly wear or remove the devices, as well as due to potential environmental factors or device calibration issues^45–47^. Previous studies have also highlighted these limitations, which should be considered when interpreting accelerometer data in sleep research.

## Conclusion

The overall findings indicate a significant lack of agreement between accelerometer measurements and parent reports of sleep duration in children. The parents’ overreporting tendency, poor diagnostic accuracy, and low levels of agreement suggest that relying solely on parental reports may not provide an accurate representation of children’s sleep duration. Future research should emphasize the importance of utilizing objective measures, such as accelerometers, to assess sleep duration in children effectively.

## Data Availability

The datasets generated during and/or analyzed during the current study are available from the corresponding author on reasonable request.
